# Evaluation of changes in choroidal thickness after implantable collamer lens surgery in high myopia patients with graves’ Ophthalmopathy (inactive phase)

**DOI:** 10.1186/s12886-020-01612-9

**Published:** 2020-08-25

**Authors:** Fanglin He, Yan Liu, Renbing Jia, Jing Zhang

**Affiliations:** 1 Department of Ophthalmology, Shanghai Ninth People’s Hospital, Shanghai Jiaotong University School of Medicine, Shanghai, China; 2 Shanghai Key Laboratory of Orbital Disease and Ocular Oncology, Shanghai, China

**Keywords:** Choroidal thickness, Graves’ Ophthalmopathy, ICL surgery, High myopia

## Abstract

**Background:**

To evaluate the safety and effectiveness of the Visian Implantable Collamer Lens (ICL) implantation in high myopic patients with inactive Graves’ ophthalmopathy (GO) by observing the changes of choroidal thickness (CT).

**Methods:**

Eight patients (16 eyes) with high myopia accompanied with inactive GO were selected as the experimental group (group A) and 18 high myopic patients (36 eyes) without GO were selected as a control group (group B). The outcomes of uncorrected visual acuity (UCVA), best spectacle-corrected visual acuity (BCVA), safety index, efficacy index, intraocular pressure (IOP), vault, corneal endothelial count, and choroidal thickness (CT) were observed. The values of CT were measured using swept-source optical coherence tomography (SS-OCT) scans.

**Results:**

The UCVA and BCVA in all operated eyes were better than that before surgery. The postoperative safety index and efficacy index were 1.23 and 1.19 in the group A, respectively, and 1.26 and 1.21 in the group B, respectively. In both groups, foveal CT increased significantly in high myopic patients at 2 h and at 3 months after surgery, compared to preoperative values. The same tendencies were observed in the inner nasal and outer nasal regions. Compared with patients without GO, the increase of CT was more obvious in GO patients, 2 h postoperatively (*P* = 0.006) and 3 months postoperatively (*P* = 0.011).

**Conclusions:**

The ICL implantation is safe and effective in high myopic patients with inactive GO. Subfoveal and nasal CT may be useful parameters for monitoring the activity of GO patients.

## Background

The Visian Implantable Collamer Lens (ICL; STAAR Surgical, Nidau, Switzerland), a posterior chamber phakic intraocular lens, is a precise, reproducible, and reversible technique for correcting refractive errors, and is especially suitable for some myopic patients with thin corneal thicknesses or corneal ectasia with limited ablation in corneal refractive surgeries. Graves’ Ophthalmopathy (GO) is an autoimmune inflammatory disorder. It is observed in approximately 25–50% of the patients with Graves’ disease (GD) and in 2% of the patients with thyroiditis [1, 2].Although GO is the most commonly observed mild clinical form, the condition of approximately 3–5% of the patients with GO worsens and progresses toward the severe form. A minority of these patients are at risk for hypophasis and corneal disorders [3]. Considering these factors, ICL surgery may be a better option for myopia patients with GO.

In past studies, the efficacy, safety and predictability of these refractive surgeries have been evaluated by the change of visual function [4] and ocular anterior segment structure [5]. The focus of myopia-related research has gradually shifted from the dioptric media of the anterior segment to the tissues of the posterior segment, such as the choroid. Choroidal thickness (CT) may alter in inflammatory diseases, such as Vogt-Koyanaghi-Harada disease [6]. In 2016, Sinan Çalişkan et al. conducted an observational cross-sectional study and showed the mean sub-foveal CT was significantly greater in GO than healthy subjects [7]. Choroid is considered to be a source of vision threatening diseases [8]. A thinner choroid may be contribute to the occurrence and progression of severe myopia-related diseases. During the recovery of myopia in chicks, the choroid compensates by getting to be thicker [9]. The thinning CT was recovered due to a reduction or neutralization of the myopiogenic stimulus to eye growth in those nearsighted children wearing overnight orthokeratology contact lenses [10]. However, a change in the CT after correcting myopic refractive errors by ICL surgery in humans, especially in patients with GO, has not been reported.

The recently developed swept-source optical coherence tomography (SS-OCT), has provide new perspectives on choroidal study, making it possible to obtain high-resolution, noninvasive cross-sectional subsurface tomographic images of biologic structures in situ and in real time. This new technique enabled us to obtain 256 raster scan images covering the entire macula to generate a CT map in a single session, and each choroidal layer was automatically detected. The aim of this study was to evaluate the potential changes in the CT after ICL surgery, using the SS-OCT technique, and to determine whether the variations of the CT correlated with GO.

## Methods

The study was approved by the Ethical Review Committees of Shanghai Ninth People’s Hospital, Shanghai Jiao Tong University School of Medicine, and conformed to the tenets of the Declaration of Helsinki.

### Patients

This cross-sectional observational pilot study included 52 eyes from 26 myopia patients who were recruited from February 2017 to November 2018 in the Department of Ophthalmology, the Ninth People’s Hospital, Shanghai Jiao Tong University of Medicine, Shanghai, China. The study was approved by the Investigational Review Board. Sixteen eyes from eight high myopia patients with a diagnosis of inactive GO without intraocular inflammation, and 36 eyes from 18 high myopia patients having no other ophthalmic or systemic diseases were included.

GO was confirmed according to the clinical manifestations (eye movements, lid-lag, diplopia and scleral show), characteristic X-ray computed tomographic images, and supportive laboratory and/or endocrine examination results. According to the clinical activity score (CAS) grading system, disease activity was calculated. We applied initial CASs, which contains only the first seven criteria [11]. Each item counts for 1 score. Patients with 3 points or more are considered as in active phase [11]. All patients with GO were in inactive phase and they were euthyroid in both clinical and laboratory examinations. They received medical treatment or radioactive iodine therapy, or underwent thyroidectomy in active GO duration ≥1 year prior to the study. None of the patients were using systemic steroids in the previous 6 months and none of the patients had decreases in visual acuity related with the GO. These patients were strongly urged to undergo the ICL surgery.

All patients were eligible for an ICL. The following inclusion criteria were used: 1) diagnosed with high myopia, the refractive diopter (D) range was from − 8.00 to-14.00 D; 2) central corneal endothelial cell count > 2000 cells/mm and anterior chamber depth > 2.80 mm; 3) a minimum of 22 years of age and was able to return for a 3-month follow-up; and 4) volunteered for the study and signed the consent form. Patients over 45 years of age with unachievable expectations, IOP > 21 mmHg, or a history of ocular surgery were excluded, as were cases with diabetes mellitus, uveitis, cataract, and other ocular or systemic autoimmune diseases that may affect the retina and CT.

All patients underwent SS-OCT, as well as a comprehensive ophthalmic examination before surgery and at 2 h, 1 day, 1 week, 1 month, and 3 months after surgery, including uncorrected visual acuity (UCVA) exams, best-corrected visual acuity (BCVA) examinations, IOP measurements, axial length (AL) determinations, slit lamp evaluations, and fundus examinations. In order to avoid the influence of diurnal variation on the CT, we obtained the OCT images at 9 a.m. and the time of operation was at 10 a.m. The first postoperative exam was 2 h after surgery, the other follow-up times were the same with preoperative examination time, and all of which were done at 9:00 a.m. Patient characteristics including age, sex, and existence of associated ocular diseases were recorded. All patients were examined by the same physician (FL He).

### SS-OCT scan protocol

**Fig. 1:**
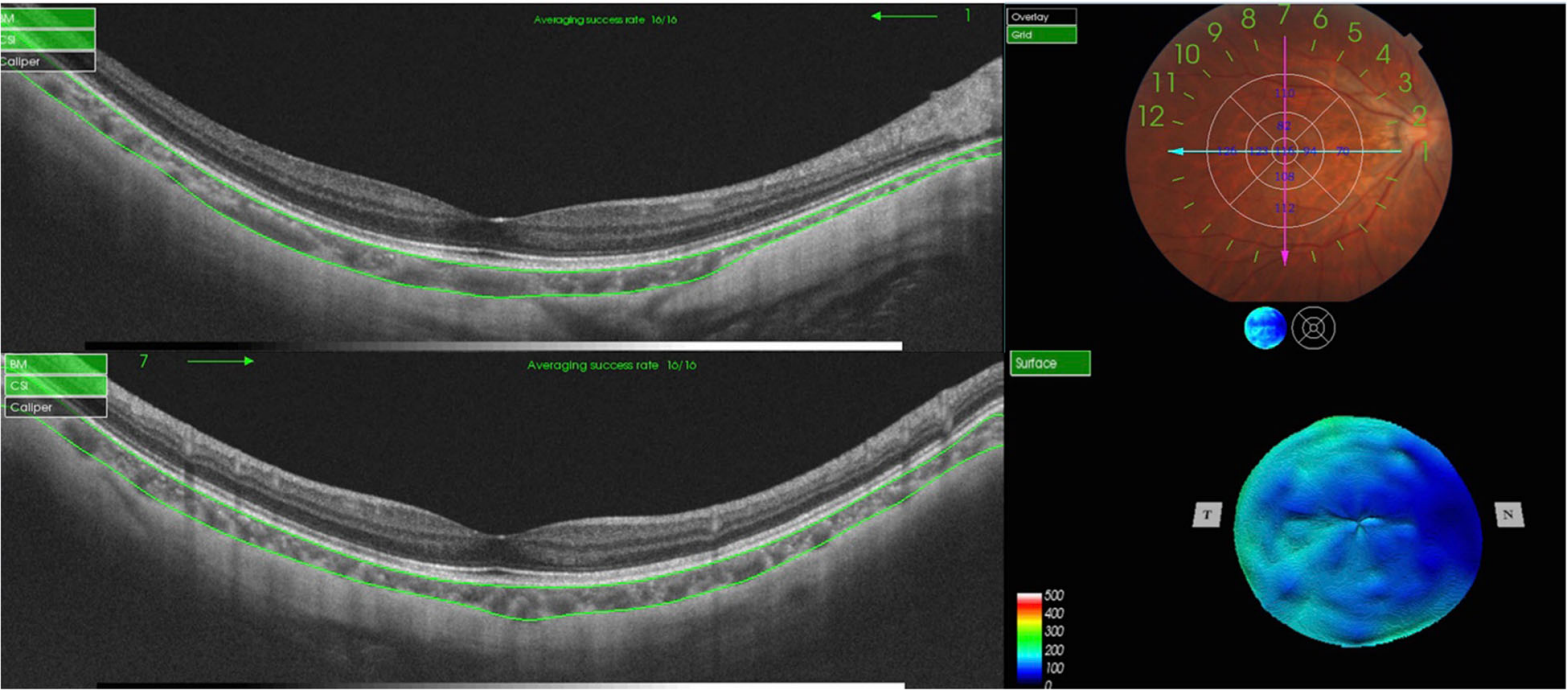
Swept-source optical coherence tomography scanning. Top and bottom left: The choroid was automatically defined as the layer between the retinal pigment epithelium and the chorioscleral interface (green line). Bottom right: CT map of the area corresponding to myopia. Top right: Study layout area and the mean regional CT calculated for the 9 sectors of the layout using the 3D macular volumetric raster scan protocol of swept-source optical coherence tomography. The units for the blue numbers are ‘μm’; BM*, Bruch’s* membrane; CSI, Chorioscleral interface; N, Nasal side; T, Temporal side.; CT, choroidal thickness

In this study, all enrolled eyes were examined with SS-OCT (DRI-1; Topcon, Tokyo, Japan), using a light source with a central wavelength of 1050 nm and a repetition rate of 1000 Hz. To get data, the 3D macular volumetric raster scan protocol, which can cover a 6 × 6 mm macular range centered on the fovea, was chosen. (Fig. 1). In previous study, we described in detail the examination method of the SS-OCT. [12] All patients were examined by two physicians (FL He and Y Liu).

### Surgical procedure

Each surgery was performed by the same experienced specialist (J Zhang). Before surgery, instillation of mydriasis agents (Tropicamide Eye Drops, Santen, Japan) was performed four times at 10-min intervals, then a 3-mm temporal corneal incision was made with a diamond knife and then the ICL V4c with a 0.36-mm central artificial hole (Hole ICL™) was inserted. A small amount of viscoelastic agent was injected in the anterior chamber after implanting the ICL. The four footplates of the ICL were placed on the ciliary sulcus behind the iris with two manipulators along the 180° axis. Viscoelastic agent was totally cleared using buffered salt solution. The ICL position was verified before the surgery was finished.

### Statistical analysis

All analyses were performed using the statistical software package GraphPad Prism (version 7.00 for Windows; GraphPad Software Inc.). Analysis of variance (ANOVA) and Pearson’s Χ^2^ test were used to compare the clinical characteristics between the two groups. CT changes after surgery in each group were assessed for by repeated-measures variance analysis (ANOVA), and independent sample *t*-tests (two groups) were applied to analyze the statistical differences between the two groups. Values are expressed as the mean ± standard deviation. *P* < 0.05 was considered to indicate a statistically significant difference.

## Results

### Clinical features of patients

#### Table 1. The clinical features of patients in the two groups

In this study, 52 eyes with high myopia had refractive D between − 8.00 and 14.00 D. The GO-high-myopia group consisted of five women and three men with a mean age of 32.57 ± 4.36 years and the no GO group consisted of 10 women and eight men with a mean age of 29.25 ± 5.06 years. The mean time of GD onset in the GO group was 6.20 ± 4.2 years.

**Table 1:** Demographic and clinical information

	Group A (GO-high-myopia patients) *n* = 8	Group B (non-GO-high-myopia patients) *n* = 18	*p*-value
Age, y	32.57 ± 4.36	29,25 ± 5.06	0.221
Gender (Female/Male)	5/3	10/8	0.668
Mean time of GD , years	6.20 ± 4.2	0	< 0.001
Preoperative SE, D	−11.84 ± 3.37	−11.21 ± 3.15 D	0.782
1w Postoperatively SE, D	−0.86 ± 0.14 D	− 0.73 ± 0.25 D	0.526
1 m Postoperatively SE, D	− 0.65 ± 0.27 D	−0.60 ± 0.57 D	0.774
3 m Postoperatively SE, D	−0.35 ± 0.27 D	−0.31 ± 0.22 D	0.812
Axial length, mm	27.79 ± 0.42	27.14 ± 1.43	0.898
IOP preoperatively, mmHg	14.35 ± 2.64	15.63 ± 1.36	0.451
IOP 2 h Postoperatively, mmHg	18.65 ± 3.42	18.72 ± 2.32	0.892
IOP 1w Postoperatively, mmHg	15.24 ± 3.11	16.07 ± 2.67	0.243
IOP 1 m Postoperatively, mmHg	15.07 ± 2.62	15.77 ± 2.91	0.455
IOP 3 m Postoperatively, mmHg	14.16 ± 3.12	15.34 ± 2.76	0.767
BCVA preoperatively,	1.42 ± 0.31	1.36 ± 0.37	0.550
BCVA 3 m Postoperatively,	−0.04 ± 0.07	−0.04 ± 0.09	0.998
UCVA 3 m Postoperatively	−0.04 ± 0.11	−0.03 ± 0.12	0.914
Safety index	1.23	1.26	0.858
Efficacy index	1.19	1.21	0.783

Values are expressed as the mean ± standard deviation or *n*. *SE* spherical equivalent of refractive error, *w* week, *m* month, *IOP* intraocular pressure, *UCVA* uncorrected visual acuity (logMAR), BCVA best-corrected visual acuity (logMAR)

The average preoperative BCVA was 1.42 ± 0.31 logarithm of the minimum angle of resolution (logMAR) (range, 1.18 to 2.0) in group A and 1.36 ± 0.37 logMAR (range, 1.16 to 2.0) in group B, respectively. The average UCVA was − 0.04 ± 0.11 logMAR (range, − 0.18 to 0.22) in group A and − 0.03 ± 0.12 logMAR (range, − 0.19 to 0.26) in group B at postoperative 3 months. The average BCVA at 3 months after the operation was − 0.04 ± 0.07 logMAR (range, − 0.10 to 0.16) in group A and − 0.04 ± 0.09 logMAR (range, − 0.14 to 0.12) in group B, which were significantly better than the preoperative BCVAs between the two groups (*p* > 0.05).

#### Safety (postoperative BCVA/preoperative BCVA)

At postoperative 3 months, in the GO group, no patients had postoperative BCVA worse than preoperative values. The GO group had the highest percentage of eyes (87.5%) with gain in lines of BCVA by one or more lines compared to group B (88.9%), respectively. Correspondingly, the safety indices (postoperative BCVA/preoperative BCVA) were 1.23 and 1.26 for groups A and B, respectively.

#### Efficacy (postoperative UCVA/preoperative BCVA)

At postoperative 3 months, the UCVA in 13 eyes (81%) was equal to or better than the preoperative BCVA in group A and 30 eyes (83) in group B. Mean postoperative UCVA (logMAR) was marginally better in group B compared to group A; however, the differences were not significant (*p* > 0.05 at all visits). Both groups showed an improvement in UCVA over time. Similar trends were observed in mean postoperative BCVA. The efficacy indices were 1.19 and 1.21, respectively.

The mean axial length (AL) was 27.79 ± 0.42 mm in group A and 27.14 ± 1.43 mm in group B. In group A, the mean spherical equivalent (SE) of refractive error was − 11.84 ± 2.37 D before surgery. One month after surgery, the SE was − 0.65 ± 0.27 D, and at 3 months postoperative, the SE was − 0.35 ± 0.27 D. In group B, the mean SE of refractive error was − 11.21 ± 2.15 D before surgery. One month after surgery, the SE was − 0.60 ± 0.57 D, and at 3 months postoperative, the SE was − 0.31 ± 0.22 D. The average washing time for both groups was 20 s. Age and sex did not differ significantly between the patient groups.

#### Intraocular pressure

The average preoperative intraocular pressures (IOPs) were 14.35 ± 2.64 mmHg (range, 9.1 to 20.0 mmHg) and 15.63 ± 1.36 mmHg (range, 10 to 20.5 mmHg) in groups A and B, respectively. Two hours after ICL implantation, the IOP was slightly increased (average, 18.65 ± 3.42 mmHg and 18.72 ± 2.32 mmHg were measured in groups A and B, respectively). There were four eyes with an IOP > 21 mmHg in the group A and seven eyes in the group B. The IOP returned to the baseline when using the lower intraocular pressure drugs after surgery. At postoperative 1 and 3 months, the IOP was stable. At postoperative 3 months, the average IOP was 14.16 ± 3.12 mmHg (range, 10.5 to 20.0 mmHg) in group A and 15.34 ± 2.76 mmHg (range, 9.6 to 20.3 mmHg) in group B, which was not significantly different from that before (and at postoperative 1 month) between the two groups (Table 1).

### CT changes

#### Table 2 And Table 3. The CT changes in group a and group B

**Fig. 2:**
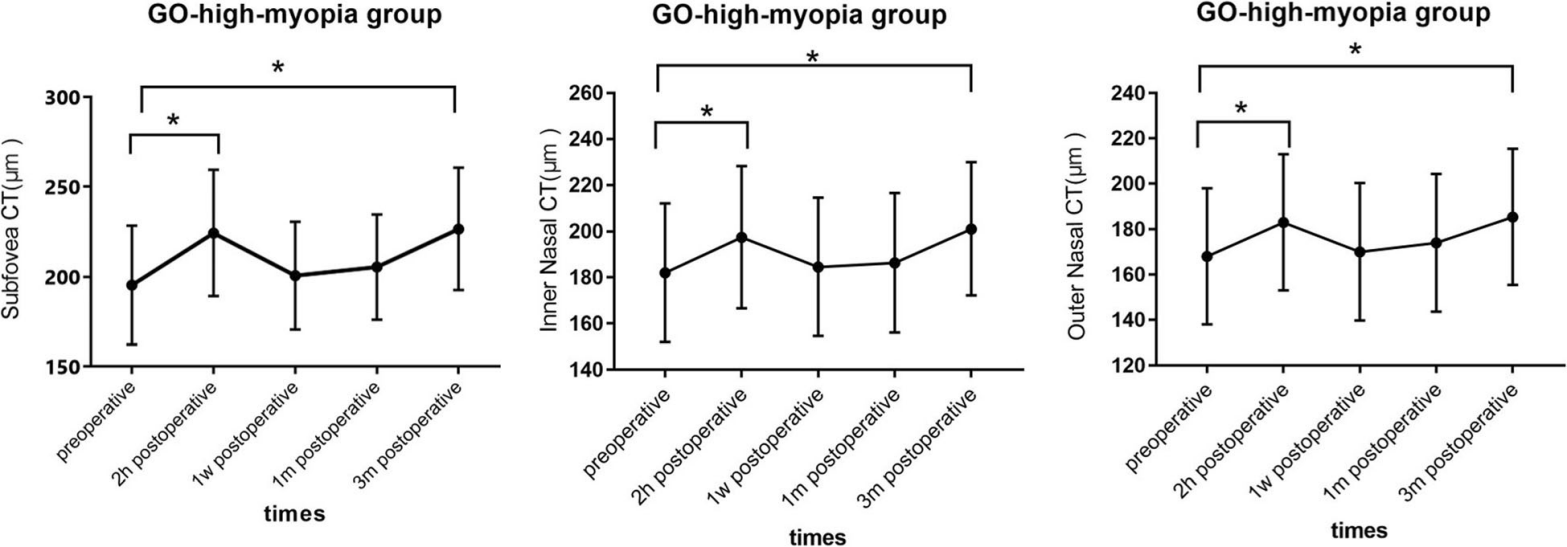
The choroidal thickness of the fovea and nasal side at different time points in GO- high-myopia group. Values are expressed as mean ± standard deviation. * *P* < 0.05. CT, choroidal thickness

Compared with the preoperative values, foveal CT increased significantly in the GO patients at 2 h after surgery (195.43 ± 34.23 vs. 224.35 ± 32.12 μm; *P* = 0.008) and at 3 months after surgery (195.43 ± 34.23 vs. 226.72 ± 31.56 μm; *P* = 0.013). The same tendency was observed in both the inner nasal CT (2 h: 182.49 ± 27.31 vs. 197.44 ± 27.19 μm; *P* = 0.042; 3 months: 182.49 ± 27.31 vs. 201.14 ± 29.36 μm; *P* = 0.018) and outer nasal CT (2 h, 168.12 ± 30.05 vs. 183.74 ± 31.45 μm, *P* = 0.042; 3 months: 168.12 ± 30.05 vs. 185.82 ± 30.07 μm, *P* = 0.036) at the same time points. Figure 2 shows the CTs of the fovea and nasal side at different time points in the GO-high-myopia group.

**Table 2:** Detailed data and statistical analysis of mean regional choroidal thicknesses for the 9 sectors in group A (GO-high-myopia patients)

Parameter	Mean (μm) ± SD												
	Pre-Operation	Post-operation				variations				*P*-value			
		2 h	1w	1 m	3 m	2 h	1w	1 m	3 m	2 h	1w	1 m	3 m
center	195.40 ± 34.23	224.35 ± 32.12	200.21 ± 32.04	205.36 ± 34.75	226.72 ± 31.56	28.92 ± 4.27.	4.78 ± 3.25	9.93 ± 4.14	31.29 ± 6.45.	**0.008**	0.967	0.668	**0.013**
TIM	205.74 ± 35.26	219.12 ± 34.29	211.35 ± 38.70	212.43 ± 30.24	220.26 ± 35.24	13.38 ± 4.23	5.61 ± 3.16	6.69 ± 2.58	14.52 ± 3,06	0.414	0.698	0.581	0.250
SIM	207.47 ± 29.74	218.39 ± 37.74	212.40 ± 36.43	214.25 ± 40.31	222.73 ± 37.54	10.92 ± 4.27	4.93 ± 6.71	6.78 ± 4.12	15.26 ± 5.33	0.357	0.784	0.699	0.177
NIM	182.49 ± 27.31	197.44 ± 27.19	184.53 ± 33.19	186.72 ± 28.35	201.14 ± 29.36	14.95 ± 7.64	2.04 ± 4.52	4.23 ± 4.35	18.65 ± 6.46.	**0.042**	0.988	0.805	**0.018**
IIM	203.43 ± 36.32	213.24 ± 34.11	204.33 ± 34.25	206.44 ± 37.18	214.35 ± 35.20	9.81 ± 5.39	0.90 ± 4.87	3.01 ± 2.56	10.92 ± 7.56	0.406	0.526	0.897	0.306
TOM	217.28 ± 41.33	227.26 ± 44.10	220.18 ± 37.32	223.65 ± 41.72	230.39 ± 24.74	9.98 ± 3.52	2.90 ± 5.13	6.37 ± 2.76	13.11 ± 6.67	0.246	0.716	0.804	0.106
SOM	208.34 ± 35.26	220.14 ± 35.67	213.22 ± 33.32	214.32 ± 34.39	221.40 ± 36.53	11.80 ± 5,23	4.88 ± 4.12	5.98 ± 3.05	13.06 ± 4.23	0.136	0.836	0.725	0.376
NOM	168.12 ± 30.05	183.74 ± 31.45	169.36 ± 29.84	174.29 ± 28.46	185.82 ± 30.07	15.62 ± 5.14	1.24 ± 4.31	6.17 ± 4.87.	17.7 ± 6.24.	**0.042**	0.998	0.708	**0.036**
IOM	204.35 ± 36.23	214.72 ± 34.36	207.53 ± 42.50	209.12 ± 33.23	216.37 ± 34.12	10.37 ± 4.23	3.18 ± 4.17	4.77 ± 4.38	12.02 ± 5.17	0.145	0.862	0.786	0.086

Values are expressed as the mean ± standard deviation. *w* week, *m* month, *NIM* nasal inner macula, *SIM* superior inner macula, *TIM* temporal inner macula, *IIM* inferior inner macula, *NOM* nasal outer macula, *SOM* superior outer macula, *TOM* temporal outer macula, *IOM* inferior outer macula

**Table 3:** Detailed data and statistical analysis of mean regional choroidal thicknesses for the 9 sectors in group B (no-GO-high-myopia patients)

Parameter	Mean (μm) + SD												
	Pre-Operation	Post-operation				variations				*P*-value			
		2 h	1w	1 m	3 m	2 h	1w	1 m	3 m	2 h	1w	1 m	3 m
center	187.24 ± 41.89	205.18 ± 40.13	187.88 ± 40.27	190.43 ± 33.47	209.32 ± 41.23	17.94 ± 5.34	0.64 ± 3.37	3.19 ± 4.07	22.08 ± 6.43	**0.031**	0.996	0.878	**0.022**
TIM	199.45 ± 36.47	207.38 ± 36.18	201.29 ± 33.27	203.18 ± 32.43	209.15 ± 36.49	7.93 ± 4.24	1.84 ± 5.26	3.73 ± 5.24	9.70 ± 3.16	0.206	0.556	0.846	0.136
SIM	196.44 ± 37.25	208.43 ± 32.59	201.71 ± 36.26	205.12 ± 34.27	211.64 ± 34.16	11.99 ± 5.14	5.27 ± 4.23	8.68 ± 3.22	15.20 ± 4.72	0.242	0.736	0.876	0.120
NIM	172.59 ± 36.25	188.60 ± 39.55	174.37 ± 35.54	176.35 ± 38.45	189.78 ± 40.27	16.01 ± 5.47.	1.78 ± 5.03	3.76 ± 6.35	17.19 ± 6.78	**0.039**	0.964	0.778	**0.030**
IIM	183.74 ± 44.18	194.26 ± 35.23	184.34 ± 36.14	187.28 ± 37.34	195.62 ± 35.46	10.52 ± 5.28	0.60 ± 5,23	3.54 ± 3.75	11.88 ± 7.14	0.106	0.946	0.846	0.087
TOM	203.24 ± 38.42	205.62 ± 36.55	203.52 ± 30.22	204.59 ± 30.77	207.16 ± 37.25	2.38 ± 4.26	0.28 ± 5.25	1.37 ± 4.29	3.92 ± 4.38	0.364	0.816	0.576	0.096
SOM	192.36 ± 43.35	201.42 ± 33.37	192.44 ± 35.27	195.55 ± 36.51	203.60 ± 36.14	9.06 ± 4.23	0.08 ± 2.64	3.98 ± 4.48	13.95 ± 6,51	0.215	0.961	0.584	0.086
NOM	153.34 ± 34.18	167.25 ± 34.76	155.38 ± 33.39	157.32 ± 34.29	171.22 ± 33.54	15.91 ± 6.55.	4.04 ± 3.52.	5.98 ± .3.26	19.88 ± 6.45.	**0.042**	0.726	0.645	**0.033**
IOM	188.44 ± 39.29	200.40 ± 34.16	193.21 ± 39.36	194.07 ± 35.95	203.47 ± 34.25	11.96 ± 2.37	4.77 ± 3.36	5.63 ± 2.87	15.03 ± 9.14	0.108	0.853	0.746	0.115

Values are expressed as the mean ± standard deviation. *w* week, *m* month, *NIM* nasal inner macula, *SIM* superior inner macula, *TIM* temporal inner macula, *IIM* inferior inner macula, *NOM* nasal outer macula, *SOM* superior outer macula, *TOM* temporal outer macula, *IOM* inferior outer macula

**Fig. 3:**
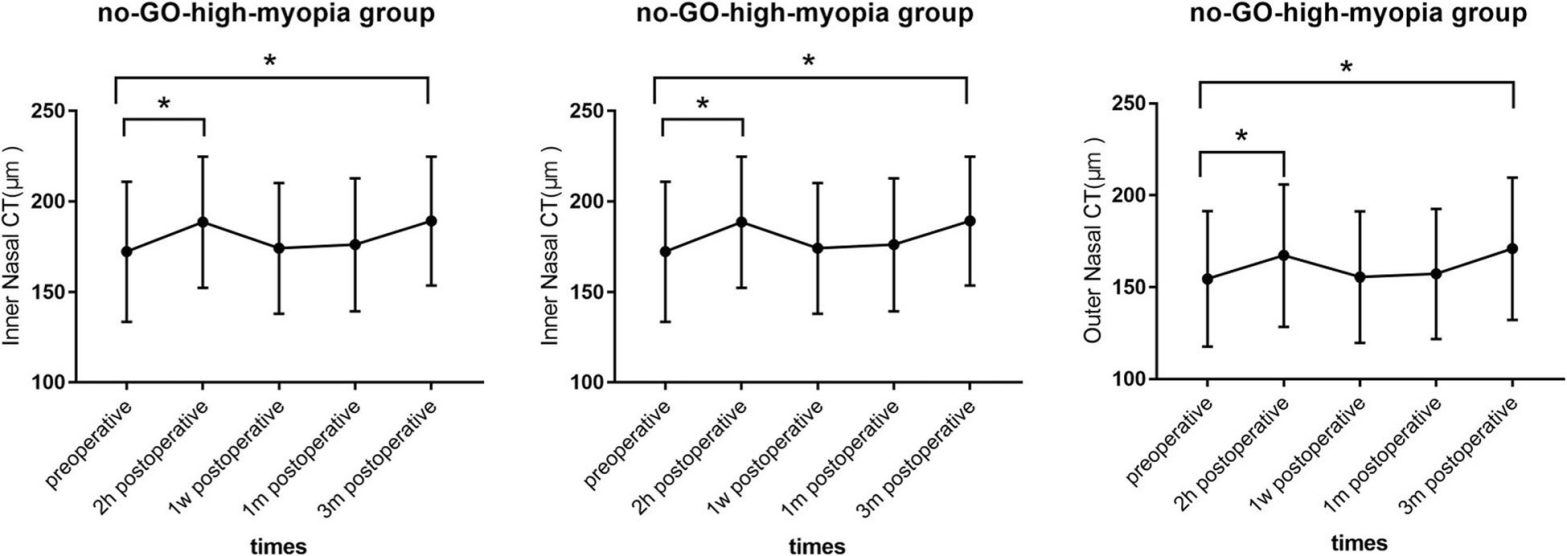
The choroidal thickness of the fovea and nasal side at different time points in no- GO- high-myopia group. Values are expressed as mean ± standard deviation. * *P* < 0.05. CT, choroidal thickness

In myopia patients with no GO, foveal CT increased significantly at 2 h and at 3 months after surgery compared to preoperative values (2 h:187.24 ± 41.89 vs. 203.18 ± 40.13 μm; *P* = 0.031; 3 months: 187.24 ± 41.89 vs. 205.32 ± 41.23 μm; *P* = 0.022), and the same tendency was observed both in the inner nasal CT (2 h: 172.59 ± 36.25 vs. 188.60 ± 39.55 μm; *P* = 0.039; 3 months: 172.59 ± 36.25 vs. 189.78 ± 40.27 μm; *P* = 0.030) and outer nasal CT (2 h: 151.34 ± 34.18 vs. 167.25 ± 34.76 μm; *P* = 0.042; 3 months: 151.34 ± 34.18 vs. 171.22 ± 33.54 μm; *P* = 0.033) at the same time points. Figure 3 shows the CTs of the fovea and nasal side at different time points in the no-GO-high-myopia group.

The mean CTs of all areas between preoperative and 1-week postoperative were similar in both groups. Between preoperative and 1-month postoperative, the mean CTs in nine regions were slightly thicker than the corresponding preoperative values, but no significant difference between the two groups was observed.

### Difference in the variation between the two groups

#### Table 4. The CT variation between the two groups

**Fig. 4:**
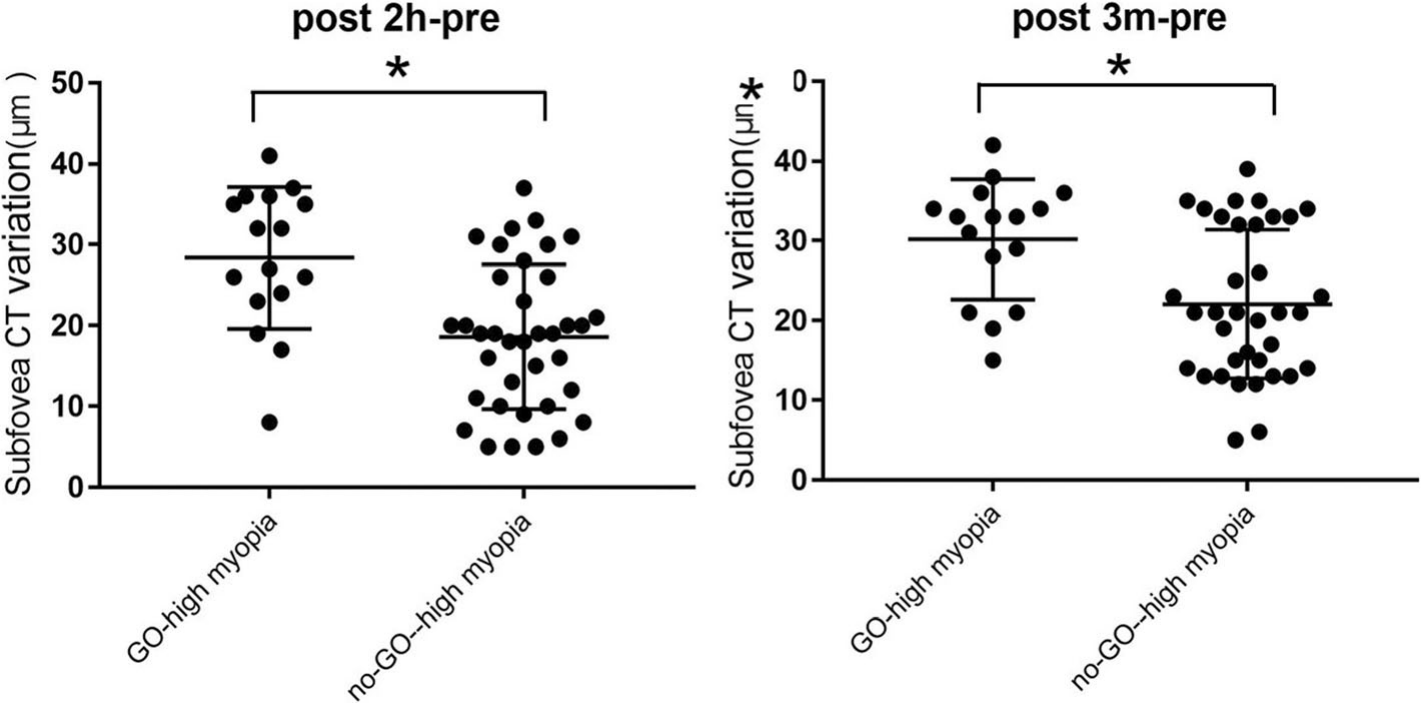
Changes in subfoveal CT in the two groups at 2 h and 3 months post-operatively vs. pre-operatively. Each data-point indicates the measurement value for one subject. * *P* < 0.05. CT, choroidal thickness

Compared with the preoperative values, subfoveal CT was significantly increased in group A patients compare to group B patients 2 h after ICL surgery (*P* = 0.006) and 3 months after surgery (*P* = 0.011). However, a statistical difference was not found in other regions between the two groups at these same time points. Figure 4 shows the significant variation distributions of each patient between the two groups.

**Table 4:** Between-group comparison of preoperative and postoperative choroidal thickness variation for the 9 sectors

Subfield	variation Mean (mm) ± SD			
	Preop to 2 h Postop	Preop to 1 W Postop	Preop to 1 M Postop	Preop to 3 M postop
Center GO-high myopia	28.92 ± 4.27.	4.78 ± 3.25	9.93 ± 4.14	31.29 ± 6.45.
no-GO-high myopia	17.94 ± 5.34	0.64 ± 3.37	3.19 ± 4.07	22.08 ± 6.43
P	**0.006**	0.306	0.105	**0.011**
TIM GO-high myopia	13.38 ± 4.23	5.61 ± 3.16	6.69 ± 2.58	14.52 ± 3,06
no-GO-high myopia	7.93 ± 4.24	1.84 ± 5.26	3.73 ± 5.24	9.70 ± 3.16
P	0.647	0.678	0.569	0.435
TOM GO-high myopia	9.98 ± 3.52	2.90 ± 5.13	6.37 ± 2.76	13.11 ± 6.67
no-GO-high myopia	2.38 ± 4.26	0.28 ± 5.25	1.37 ± 4.29	3.92 ± 4.38
P	0.565	0.734	0.521	0.068
NIM GO-high myopia	14.95 ± 7.64	2.04 ± 4.52	4.23 ± 4.35	18.65 ± 6.46.
no-GO-high myopia	16.01 ± 5.47.	1.78 ± 5.03	3.76 ± 6.35	17.19 ± 6.78
P	0.654	0.748	0.898	0.736
NOM GO-high myopia	15.62 ± 5.14	1.24 ± 4.31	6.17 ± 4.87.	17.7 ± 6.24.
no-GO-high myopia	15.91 ± 6.55.	4.04 ± 3.52.	5.98 ± .3.26	19.88 ± 6.45.
P	0.982	0.661	0.948	0.762
SIM GO-high myopia	10.92 ± 4.27	4.93 ± 6.71	6.78 ± 4.12	15.26 ± 5.33
no-GO-high myopia	11.99 ± 5.14	5.27 ± 4.23	8.68 ± 3.22	15.20 ± 4.72
P	0.154	0.806	0.712	0.920
SOM GO-high myopia	11.80 ± 5,23	4.88 ± 4.12	5.98 ± 3.05	13.06 ± 4.23
no-GO-high myopia	9.06 ± 4.23	0.08 ± 2.64	3.98 ± 4.48	13.95 ± 6,51
P	0.738	0.567	0.714	0.893
IIM GO-high myopia	9.81 ± 5.39	0.90 ± 4.87	3.01 ± 2.56	10.92 ± 7.56
no-GO-high myopia	10.52 ± 5.28	0.60 ± 5,23	3.54 ± 3.75	11.88 ± 7.14
P	0.747	0.833	0.957	0.832
IOM GO-high myopia	10.37 ± 4.23	3.18 ± 4.17	4.77 ± 4.38	12.02 ± 5.17
no-GO-high myopia	11.96 ± 2.37	4.77 ± 3.36	5.63 ± 2.87	15.03 ± 9.14
P	0.753	0.729	0.852	0.845

Values are expressed as the mean ± standard deviation. *w* week, *m* month, *NIM* nasal inner macula, *SIM* superior inner macula, *TIM* temporal inner macula, *IIM* inferior inner macula, *NOM* nasal outer macula, *SOM* superior outer macula, *TOM* temporal outer macula, *IOM* inferior outer macula

There was no statistically significant difference in the CT variation in each region between the two groups at 1 week. The CT variations between preoperative and 1-month postoperative eyes were also similar in the two groups.

## Discussion

The global prevalence of GO is about 0.1–0.3% [1], which can be seen as exophthalmos, upper eyelid retraction, restrictive strabismus, diplopia and other symptoms, and even lead to optic neuropathy, such as the threat of visual acuity lesions [13, 14]. How to solve a series of complications and how to improve the quality of life of GO patients have been of increasing interest to medical workers. At present, clinicians can effectively control the progress of GO patients through the use of drugs, radiotherapy, orbital decompression and other means [15]. However, how to solve the refractive correction problem of GO patients with high myopia has always been difficult, especially for some GO patients who do not want to endure wearing heavy frame glasses. Because GO can easily cause corneal infection and other problems, corneal refractive surgery will make corneal thicknesses thinner, resulting in a decline in corneal disease resistance. There has been controversy regarding corneal refractive surgery for GO patients with high myopia. Thus, for myopic patients with inactive GO, an ICL implantation is preferred. Previous studies demonstrated the implantation of ICL was an effective surgical treatment for the correction of refractive errors, showing good optical quality [16, 17], especially using the new generation of ICL V4c, which appears to be better than the conventional ICL and shows significant improvements in aberrations, postoperatively [18, 19]. With the increasing use of ICLs worldwide, there is growing concern about the impact of the operation. However, no data exist regarding the possible effects of ICL surgery on the choroid, particularly for inactive GO.

In the present study, we used the latest model of ICL4 to solve refractive problems of patients with inactive GO. Compared with non-GO patients, there was no difference in the safety and efficacy indices of inactive GO patients (Table 1). Our study reported that the choroid became thicker following ICL surgery in high myopic patients with inactive GO, and the changes of CT in inactive GO patients were obviously greater than that of non-GO patients. CT can also be altered in thyroid-associated ophthalmopathy because of a decrease of choroidal blood flow of more than 70%. We suggest that the choroid layers of these people could be more sensitive to surgery, even though they are already in a GO inactive phase. CT may be a useful parameter to monitor disease activity and allow us to judge the interaction between GO and ICL surgery by measuring the CT changes before and after surgery. Automatic detection using SS-OCT can clearly identify the deep structures and provide high resolution images, providing a revolutionary technology for the determination of CT. Interestingly, we found that only the changes in the fovea and nasal CT were statistically significant, indicating that the minimum values measured around the central fovea were not sufficient to assess the whole macula.

The effect of various refractive surgeries on CT has been reported in recent years [20, 21]. Previous studies have shown that CT enhancement at 1 month after surgery was statistically significant [22]. This was not consistent with our results, which showed a peak at 3 months; but the trend of postoperative thickening was consistent. According to the present results, the CTs showed a tendency to become thicker after the refractive error corrected. The proposed physiological mechanisms of dynamic increase or decrease of choroidal thickness include contraction and relaxation of nonvascular smooth muscle [23], fluid redistribution caused by osmotic changes [24], and changes of choroidal blood flow [25, 26].

It is known that surgical trauma can lead to the release of prostaglandins in the aqueous humor, thus causing damage to the blood/aqueous barrier. Our study found that CTs became significantly thicker 2 h after surgery, especially in the fovea. We believe that this was related to inflammation and vascular permeability after surgery. These effects can lead to inflammation medium (e.g., endotoxin and immune complex) spread to the vitreous and reach the retina, causing internal/blood retinal barrier burst, and leads to the secretion of inflammatory mediators cascade and the increase of capillary permeability [27]. Previous research found that an obvious inflammatory response induced by surgical trauma would account for the acute inflammatory gene transcriptions observed in the retina [28, 29]. They found the surgical trauma induced an obvious inflammatory response, which would account for the acute (30 min after surgery) inflammatory gene transcriptions observed in the retina. We hypothesize that similar changes may occur in ICL implantation, because the ICL implantation and cataract surgery are both intraocular surgeries accompanied by the destruction of blood / aqueous barrier. In this operation, we improved several surgery steps: no viscoelastic agent was injected before the ICL was implanted, only a small amount of viscoelastic agent was injected after the ICL was implanted, and the time to wash the viscoelastic agent was also reduced to 10s. In this way, the interference to the intraocular environment can be relieved, and the use and residue of viscoelastic agent can be reduced to prevent intraocular pressure rise after surgery. The intraocular pressure measured 2 h after surgery did, however, increase by about 3–5 mmHg, compared to that before surgery. When the IOP increases to a certain extent, it may affect the choroidal blood flow, thus affecting the thickness of the choroid [30]. Our study was limited in that we could not measure the choroidal blood flow at that time. The results showed that the CT was slightly thickened 2 h after ICL implantation. We speculated that the inflammatory mechanism played a leading role in the early stage.

In addition to the above, the accommodation was also involved in the change in the CT. High myopia has a lag in accommodation, thus resulting in a low accommodation reserve. According to previous studies, CT changes induced by visual defocus are reversible [24]. In this study, we found that subfoveal CTs in both groups slightly increased in 1 week and 1 month after ICL implantation. As shown in Table 2 and Table 3, similar results were found in the nasal area. It is suggested that CT has a recovery period with time. In the next month after surgery, the choroid gradually recovered, which should be related to the gradual regression of intraocular inflammation and vascular permeability. This indicated the degree of CT induced by surg surgical trauma was weakly and inversely related in the short-term. In the current study, we also found that the increase in subfoveal CT approached its maximum at postoperative 3 months; there was a marked improvement in visual quality. Previous studies have speculated that in the absence of a clear retinal image, ocular accommodation may be induced [31–33], We postulated that 3 mo after the operation, the corrected refractive errors in retinal imaging quality can achieve the most clear and stable level, thereby decreasing the need for accommodation. We also found that in the choroidal map that significant changes in CT existed only in nasal regions except for the thickening of the foveal CT. The mechanism is not clear, but it may be related to accommodation, which is mainly regulated by ciliary muscle and ligament of lens [23]. Eyeball convergence and contraction of medial rectus muscle may be one of the factors of obvious changes in nasal choroid [34].

Several factors may influence the increase in subfoveal CT in GO patients. Elevated suprascleral venous pressure value has been demonstrated in GO [35]. The increase in venous pressure may lead to an elevation of IOP. Some studies have shown that CT might be affected from the venous obstruction and congestion in these patients [36]. Odrobina et al. found that after scleral buckling surgery using an encircling band, in long-term observation, subfoveal CT of eyes was significantly thicker [37]. Shinohara et al. reported that the choroidal thickness of a patient with carotid cavernous fistula (CCF) was increased secondary to the superior orbital vein (SOV) congestion [38]. They suggested that it may cause may lead to increased choroidal pressure and may increase the subfoveal CT. In the present study, as show in Table 4, we observed that there were significant differences in subfoveal CT variations between the two groups. Although in the two groups, the degree of intraocular pressure change is similar, patients with GO were more sensitive to intraocular operation, and intraocular surgery is more likely to cause choroidal blood flow fluctuation and change vascular permeability in patients with GO, resulting in obvious choroidal thickening. The elevation of the CT might be an early sign of venous congestion that occurs before the elevation of IOP. As mentioned above, the choroidal infiltration of inflammatory cells, increased exudation, increased vascular leakage, and ocular blood flow alterations may lead to the changes in CT [39]. We speculate that the surgery affected the CT by activating these inflammatory cells and factors in the eyes, triggering potential inflammation or an immune response. Which may be why CTs in GO patients are more sensitive to the ICL surgery.

There were several limitations in our study. The first was that our study was limited by the relatively short follow-up period, so it is not known how long the effects may last once the refractive error is corrected, this requires a long-term follow-up involving a larger sample. We also have no detailed comparison of the potential effects of diopter differences at different times after surgery.

## Conclusion

we observed that CT significantly increased 2 h after surgery, reaching a peak at 3 months after surgery, especially in the subfoveal and temporal areas. CTs increased significantly in the GO-high-myopia group, and CT preceded the occurrence of many retinal abnormalities. In the future, we hope that more attention will be focused on the thickening of the CT after ICL surgery. These data could enable us to better evaluate the postoperative safety of GO patients with high myopia.

## Data Availability

The data used to support the findings of this study are available from the corresponding author upon request.

## Abbreviations

ICL: Implantable Collamer Lens
GO: Graves’ ophthalmopathy
UCVA: Uncorrected visual acuity
BCVA: Best spectacle-corrected visual acuity
IOP: Intraocular pressure
SS-OCT: Swept-source optical coherence tomography
CAS: Clinical activity score
SOV: Superior orbital vein
GD: Graves’ disease
